# Examination of the Effects of a Play-Based Mindfulness Training Program on Resilience, Emotion Regulation Skills, and Executive Functions of Preschool Children

**DOI:** 10.3390/children13010110

**Published:** 2026-01-12

**Authors:** Betül Kapkın İçen, Osman Tayyar Çelik

**Affiliations:** 1 Child Development Program, Health Services Vocational School, Inonu University, Malatya 44050, Turkey; 2 Faculty of Health Sciences, Department of Child Development, Inonu University, Malatya 44050, Turkey; otayyar.celik@inonu.edu.tr

**Keywords:** emotion regulation, preschool children, play-based training, resilience, executive function, mindfulness

## Abstract

Background/Objectives: The cognitive processes underlying learning are critical for educational practices. While mindfulness-based approaches to strengthening these cognitive processes have become widespread, studies focusing on game-based development of executive functions, particularly in preschool settings, are limited. The primary objective of this study is to develop a play-based mindfulness intervention program for preschool children and to examine the effects of this program on preschool children’s resilience, emotion regulation skills, and executive functions. Methods: The study employed a pretest–post-test control-group experimental design. The study group consisted of 40 children (20 experimental and 20 control) aged 5–6 years, attending a kindergarten in Malatya province, Türkiye. The Devereux Early Childhood Assessment Scale (DECA-P2), Emotion Regulation Scale (ERS), and Childhood Executive Functions Inventory (CHEXI) were used as data collection tools. Independent-samples *t*-tests were used for baseline analysis, and a two-way repeated-measures ANOVA was used to evaluate the program’s effects. Results: Findings showed that there was a statistically significant difference between the pre-test and post-test mean scores of the children in the experimental group compared with those in the control group for resilience, emotion regulation, and executive function (*p* < 0.05). Conclusions: Strong evidence was obtained that play-based mindfulness training has positive effects on the cognitive and emotional development of preschool children.

## 1. Introduction

The preschool period, an important phase of early childhood, is when an individual’s permanent life skills begin to develop and when development in social, emotional, cognitive, language, and motor areas occurs most rapidly. Experiences gained during this period directly affect an individual’s resilience, personality structure, social relationships, and motivation to learn [1]. There is strong evidence that quality early childhood education improves motor function, language proficiency, and cognitive function, including social outcomes [2,3]. During this critical period, holistic, structured programs that meet children’s developmental needs are needed. Rather than approaches aimed solely at enhancing cognitive development, programs that also encompass social skills, resilience, emotional awareness, attention management, and stress management are gaining prominence. Indeed, better social and emotional skills and behavioral development in early childhood are also linked to later academic success and well-being [4,5,6]. This critical period is characterized by a marked acceleration in executive functions, emotional regulation skills, and social problem-solving, making it a period sensitive to educational and preventive interventions [7,8]. In this context, there is growing interest in mindfulness interventions to support children’s skills in the early years [9,10].

There is a substantial body of literature on the effectiveness of mindfulness practices as an intervention in both clinical and non-clinical adult and adolescent samples [11,12]. Growing evidence on the effectiveness of mindfulness-based interventions has encouraged researchers to develop mindfulness-based intervention programs for younger age groups. Recent studies with preschool children report that mindfulness-based programs support executive functions [13], improve mental health [14], significantly enhance motor skills, and reduce behavioral difficulties [15]. Despite this evidence, there remains a lack of understanding of how to integrate mindfulness-based interventions and programs into early childhood education [16]. This research aims to examine the multifaceted effects of the program on preschool children’s social-emotional and cognitive skills by integrating play into the mindfulness program as an element that triggers children’s emotions, intelligence, and exploratory experiences [17].

### 1.1. Mindfulness and Play in Early Childhood

The concept of mindfulness has its roots in Buddhist traditions dating back approximately 2500 years. Today, however, it is known as a more secular concept, not directly related to any religious or philosophical system [18]. In particular, Kabat-Zinn’s (2023) definition of mindfulness as the ability to pay attention to one’s experience in any field without judgment has accelerated its consideration in scientific studies [19]. Brown and Ryan (2003) define mindfulness as a state of being aware of what is happening in the moment and consider it a skill that can be learned through regular practice [20]. While early studies focused primarily on adult samples, research over the past decade has increasingly expanded to include primary school and older students [21]. Studies conducted with older children have reported a range of positive outcomes. These studies [12,22,23] report that mindfulness programs reduce internalizing problems and promote well-being among students, improve emotion regulation skills, and positively impact academic achievement and well-being. Evidence that mindfulness interventions support students’ mental health and wellbeing has encouraged the development of these programs for younger age groups.

It is claimed that early childhood is an ideal time to develop awareness skills [24]. Research with preschool children has shown that mindfulness programs improve executive functions [13,25], self-regulation and problem behaviors [26,27], prosocial behavior and perspective-taking [9], emotion regulation and well-being [14,16,28]. However, there are increasing criticisms regarding the duration, content, and structure of these programs [29,30]. In this context, programs based on short, concrete, and gamified activities that encourage curiosity are recommended as strategies to increase attention in preschool children [21,29].

It is well known that play contributes to the cognitive development of pre-school children [31]. Furthermore, play is the fundamental language and tool for learning in early childhood. Children explore their environment through play, try out social roles, express their emotions, and learn by experiencing problem-solving and negotiation skills. Therefore, designing intervention programs implemented in early childhood to be play-based is considered critical for both developmental appropriateness and a learning process embedded in a natural setting with high intrinsic motivation [32,33]. In this context, as play is one of the most effective learning tools during the preschool period, a play-based approach is also important for fostering mental awareness. Play, a way for children to express themselves, provides a natural learning environment for developing skills such as social role-taking, cognitive flexibility, empathy, and emotional regulation [34,35]. Hirsh-Pasek and Golinkoff (2006) stated that play-based learning, with its experiential and exploratory nature, supports children’s attention control, self-regulation, and communication skills [36]. Play-based mindfulness activities help children develop mindfulness skills in fun, concrete ways. For example, activities such as practicing breathing exercises with toys or recognizing emotions through facial expressions are developmentally appropriate for young children. Through these activities, children develop mindfulness skills and make progress in related areas such as resilience, executive functions, and emotion regulation.

### 1.2. Resilience in Early Childhood

Resilience is the capacity of an individual to adapt and recover flexibly in situations of trauma, stress, crisis, and adversity [37]. However, resilience is not merely an individual characteristic from a developmental perspective; it is also considered the capacity to successfully adapt to challenges that threaten a system’s function, existence, or future development. Therefore, a child’s adaptive capacity is also shaped by their relationships with their environment and environmental resources [38]. In early childhood, resilience develops in parallel with rapid brain and socio-emotional development; it is strengthened by self-regulation-based skills such as emotion regulation, impulse control, sustained attention, stress coping, and social relationship formation [39]. This skill, developed at an early age, is seen as a protective factor for an individual’s success in school life, sustainability in social relationships, and future mental health [40]. Additionally, studies in the Turkish context [41,42] reveal that basic emotional and social skills, particularly emotional competence and empathy, are associated with preschool children’s resilience and coping skills; furthermore, some studies indicate age- and gender-related variations. Furthermore, there is evidence that mindfulness-based programs can contribute to resilience processes by strengthening children’s stress-coping, acceptance of internal experiences, self-compassion, and emotional awareness [10,43]. Mindfulness-based programs enable children to recognize sources of stress, helping them build emotional resilience in stressful situations and develop more conscious responses [44]. Resilience, which develops children’s capacity to cope with adversity and stress, has a holistic structure with emotion regulation skills that complement the process.

### 1.3. Emotional Regulation in Early Childhood

Emotion regulation is often addressed within the broader framework of self-regulation, a multidimensional construct encompassing emotional, behavioral, and cognitive regulation [29]. Emotion regulation is defined as the totality of internal and external processes aimed at monitoring, evaluating, and modifying emotional responses to enable the individual to achieve their goals and maintain social interactions by adapting to environmental demands [45,46,47]. In early childhood, emotion regulation is one of the key determinants of social-emotional and academic development; awareness of emotion regulation strategies becomes particularly evident between the ages of 3 and 5, with emotion socialisation, shaped by parental and teacher responses, playing a critical role in this development [48]. Children initially use more external/behavioral strategies (e.g., distraction, seeking help), but as they get older, they turn to more internalized and cognitive strategies [49,50]. Emotion regulation skills increase with age, but individual differences and gender effects may be observed; however, gender-related findings are sensitive to strategy type and context [47]. While these skills are closely related to school readiness, social adjustment, and academic functioning [51], deficiencies may increase the risk of internalized and externalized behavioral problems in later periods [52,53]. Overall, findings indicate that emotion regulation is strongly linked to outcomes such as peer relationships, classroom adjustment, and engagement in learning; increased emotion-focused instruction and emotion coaching practices in the classroom may be associated with lower negative emotion expression and more adaptive emotion-related behaviors [48,54]. Through mindfulness-based programs, children recognize their emotions without suppressing them, accept them without judgment, and develop the capacity to cope with emotions in healthy ways [55]. For example, a child who can calm themselves by taking deep breaths when angry can also control their impulsive behavior. Mindfulness, which forms the cornerstone of emotion regulation, also supports a child’s social functioning and self-awareness [8]. In conclusion, effective emotion regulation is decisive not only in social relationships but also in cognitive processes, and it forms a critical foundation, especially for the development of executive function skills.

### 1.4. Executive Functions in Early Childhood

Executive functions encompass many cognitive skills, including planning, cognitive flexibility, attention control, inhibition, task management, and working memory [56]. These skills are fundamental to children’s compliance with rules in social settings, their active participation in learning processes, and task completion behaviors. Diamond (2013) stated that executive function skills develop rapidly during the preschool period, and that interventions during this period can have lifelong effects [57]. For example, a child with poor inhibitory skills may have difficulty lining up or waiting their turn to speak. In contrast, a child with poor planning skills may have difficulty organizing a task from start to finish. Executive functions are positively associated not only with academic domains but also with emotion regulation, prosocial behaviors, peer acceptance, and cooperative classroom behaviors; they are negatively associated with internalization, externalization, and attention problems [58,59]. The level of executive functions is also linked to biological and environmental factors such as language development, early communication gestures, socioeconomic status, parenting styles, and physical activity, which places executive functions in a central position for both cognitive and socio-emotional development [60,61,62]. With mindfulness practices that support executive function skills, children can maintain attention, inhibit inappropriate behaviors, and develop alternative perspectives on events [63].

Considering that all these areas exhibit a holistic and interactive structure, it is seen that play-based mindfulness programs provided in early childhood play a supportive role not only in a single skill area but also in the child’s overall development [36]. However, a significant portion of the studies in this area focus only on a single variable, such as emotion regulation, executive functions, or resilience. Experimental studies addressing these three variables simultaneously are limited in the literature [63]. A recent study by Aydın and Özbey (2022) [64] experimentally examined the effect of a mindfulness-based program on executive function in preschool children. In this study, the implemented program was found to significantly improve executive function areas, including planning skills, attention control, and working memory [64]. Similarly, Canol (2021) found that a self-regulation-based training program increased children’s social skills and self-control [65]. However, in these studies [19,20], due to the nature of the pre-school period, a play-based pedagogical framework has not been explicitly and systematically incorporated into the design.

Therefore, this research offers a unique contribution to the field by holistically and experimentally examining the effects of a play-based mindfulness program on interrelated yet distinct developmental domains, such as resilience, emotion regulation, and executive functions. Furthermore, this research adds nuance to the field by integrating play, which has a unique contribution to supporting preschool children, into a mindfulness-based intervention program. In this context, the aim of this study is to develop a play-based mindfulness intervention program for preschool children and to examine the effects of this program on preschool children’s resilience, emotion regulation, and executive function skills. In line with this general purpose, answers to the following questions were sought:

1. Does a play-based mindfulness intervention program improve resilience in preschool children?
2. Does a play-based mindfulness intervention program improve emotion regulation skills in preschool children?
3. Does a play-based mindfulness intervention program improve executive functions in preschool children?

## 2. Materials and Methods

This study has two main objectives: (1) to develop a play-based mindfulness intervention program specifically for preschool children and (2) to examine the effectiveness of this program through a small-scale experimental design. Accordingly, the study employed a two-group pre-test–post-test design including an experimental group and a control group. Following baseline assessment, the experimental group received the play-based mindfulness intervention, whereas the control group continued routine preschool education. Outcome measures were administered at pre-test and post-test to evaluate changes in resilience, emotion regulation, and executive functions.

### 2.1. Participants

**Figure 1:**
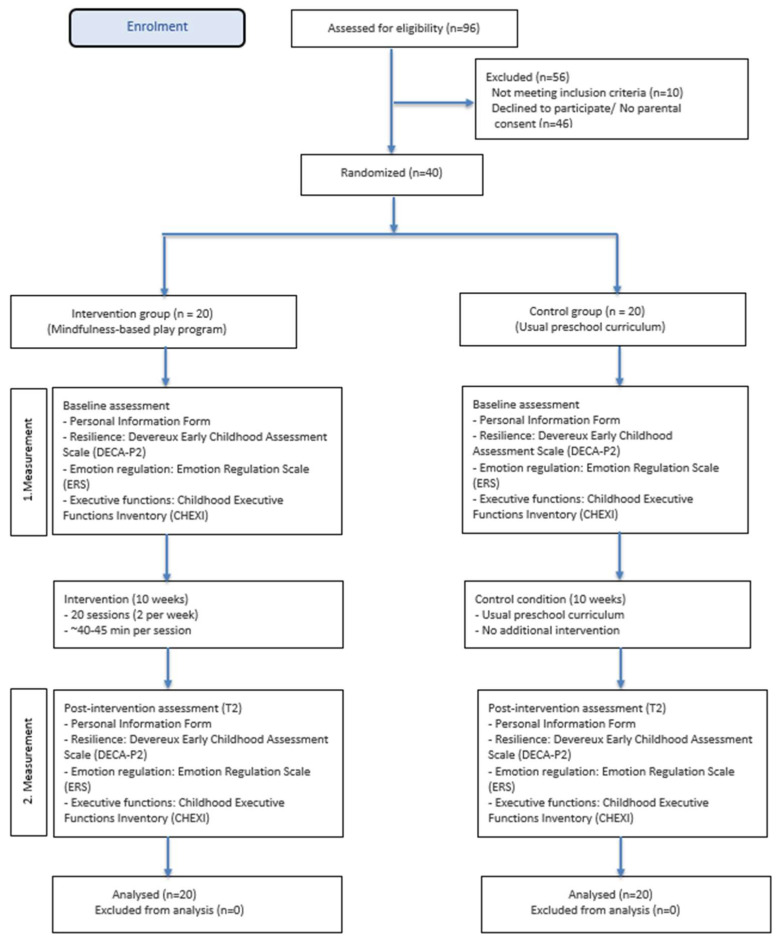
Flow chart of the study.

This study has two main objectives: (1) to develop a play-based mindfulness intervention program specifically for preschool children and (2) to examine the effectiveness of this program through a small-scale experimental design. The study group consisted of children aged 5–6 attending a preschool affiliated with the National Education Directorate in Malatya, Türkiye. The sample size was determined via an a priori power analysis in G*Power 3.1.9.4. In the planning phase, we initially estimated power based on between-group differences, using an expected effect size of Cohen’s f = 0.41 derived from a comparable intervention targeting executive functions (Thierry et al., 2016) [26]. With α = 0.05, 1 − β = 0.80, 2 groups, and 2 measurements (pre-test–post-test), this initial analysis indicated that approximately 19 participants per group would be sufficient; therefore, the study was planned with 20 participants per group. Following reviewer/editorial feedback, we report power for the effect of primary interest, namely the time × group (within–between) interaction. Accordingly, the analysis was recalculated using F tests: “ANOVA: Repeated measures, within–between interaction.” Using the same effect size (f = 0.41) [26], α = 0.05, 1 − β = 0.80, 2 groups, 2 measurements, correlation among repeated measures = 0.50, and ε = 1, the required total sample size was 14. The final sample comprised 40 participants (20 per group), thus exceeding the calculated minimum. Figure 1 summarizes participant flow through the study following CONSORT guidelines.

The study group was formed using criterion sampling, a purposive sampling method. Criterion sampling is based on the selection of predetermined situations, individuals, or events that possess certain characteristics. This method aims to obtain high-quality data relevant to the study’s purpose [66]. Certain characteristics were considered as criteria in determining the experimental and control groups. This included the children’s typically developing status, their lack of special needs, and their lack of prior play-based mindfulness training. Furthermore, the willingness of teachers and families to participate in the study, the children’s families’ similar socioeconomic status, and a balanced distribution of children were among the key criteria.

Participants were selected according to the specified criteria and randomly assigned to two equal groups: the experimental group (n = 20) and the control group (n = 20). Detailed information on the participants’ demographic characteristics is presented in Table 1. First, an examination of the gender distribution reveals a balanced representation of boys and girls in both groups. In the experimental group, girls constituted 45% (n = 9) and boys 55% (n = 11), while in the control group, girls constituted 50% (n = 10) and boys 50% (n = 10). This finding demonstrates a homogeneous gender distribution across the groups. Overall, the demographic characteristics of the experimental and control groups were largely similar, with minor differences concentrated primarily in the variables of parents’ occupational status and number of children. These distributions indicate that there are no statistically significant differences between the study groups in terms of sociodemographic characteristics (*p* > 0.05).

### 2.2. Measures

#### 2.2.1. Personal Information Form

The Personal Information Form, prepared by the researchers, was used to collect information on the parents and children of the experimental and control groups that comprised the study sample. The parents completed the form. It included questions regarding the children’s gender and age, whether the parents were alive, the mother’s educational level, the father’s educational level, the number of children, and the mother’s and father’s occupations.

#### 2.2.2. Devereux Early Childhood Assessment for Preschoolers (DECA-P2)

DECA-P2, developed by LeBuffe and Naglieri (2013) and adapted into Turkish by Atabay Demir and Sönmez (2020), was used to assess the resilience of children aged 5–6 [67,68]. DECA-P2 was developed to assess the social and emotional development and well-being of preschool children and can be completed by children’s teachers, early childhood professionals, or caregivers. Recent international studies have also shown that this scale is used as a valid and reliable tool in assessing children’s resilience [69,70]. The scale includes 38 items rated on a 5-point Likert-type scale (0: Never–4: Very often). DECA-P2 consists of two main scales: Total Protective Factors (TPF; 27 items) and Behavioral Concerns (11 items) [67,68]. The TPF scale comprises three subscales: Self-Regulation (9 items), Attachment/Relationships (9 items), and Initiative (9 items). As in previous studies [69,70], this study also used three subscales of the Total Protective Factors (TPFs) (Self-Regulation, Attachment/Relationships, and Initiative) to measure the resilience of preschool children. Higher scores on these subscales indicate higher resilience. In the Turkish adaptation study, the scale’s Cronbach’s alpha coefficient was calculated as 0.91 for the teacher form. For the subscales, the values were: Self-regulation = 0.86, Attachment/Relationship = 0.76, and Initiative = 0.88. In the present study, the Cronbach’s alpha coefficients were calculated as 0.75 for Self-regulation, 0.80 for Attachment/Relationship, and 0.78 for Initiative.

#### 2.2.3. Emotion Regulation Scale (ERS)

The Emotion Regulation Scale was developed by Shields and Cicchetti (1997) and adapted into Turkish by Batum and Yağmurlu (2007) [71,72]. The scale has been used as a valid and reliable measurement tool in studies to assess children’s emotion regulation skills in the preschool period [22,23]. The scale consists of 24 items and two subscales to assess preschool and school-age children’s emotional responses and how these emotions are regulated and expressed in accordance with environmental conditions. The subscales are as follows: Emotion Regulation and Variability/Negativity (hereafter referred to as Negativity). The Negativity subscale measures the instability of the child’s emotional responses and the tendency to negative emotions (e.g., “Moods are very variable; easily frustrated and irritated.”). The Emotion Regulation subscale assesses the child’s ability to express emotions appropriately in the environment (e.g., “May say that he/she is sad, angry, or frightened.”). Teachers or parents rate how often the child exhibits the behaviors on the list by scoring them from 1 (never) to 4 (always). In the adaptation study, the scale’s factor structure, validity, and reliability properties were retested on a Turkish sample. In this context, the scale’s Cronbach’s alpha coefficient was reported as 0.75 for assessments made by mothers and 0.84 for assessments made by teachers [72]. In this study, teachers assessed the emotion regulation skills of children in the experimental and control groups, and the scale’s Cronbach’s alpha coefficient was 0.80.

#### 2.2.4. Childhood Executive Functioning Inventory(CHEXI)

In this study, the Childhood Executive Functioning Inventory(CHEXI) (48–72 months), developed by Thorell and Nyberg (2008) and adapted to Turkish by Arslan Çiftçi et al. (2022), was used to assess children’s executive function skills [73,74]. The scale consists of two subscales: 13 items for “working memory” and 11 items for “inhibition”. The CHEXI is intended to assess children’s ability to sustain attention, remember instructions, and control their behavior. For example, statements such as “Has difficulty remembering long instructions” or “Has difficulty ending an activity after being told to stop” help observe difficulties in children’s executive function processes. Participants (teachers or parents) rate the extent to which each behavior applies to the child by scoring it from 1 (definitely not true) to 5 (definitely true). Higher scores on the scale indicate difficulties in executive function. In the Turkish validity and reliability study, the scale demonstrated high internal consistency. Cronbach’s alpha coefficients were reported as 0.95 for the working memory subscale and 0.91 for the inhibition subscale [74]. In this study, Cronbach’s alpha coefficients were calculated as 0.86 for the working memory subscale and 0.88 for the inhibition subscale.

### 2.3. Procedure

Before implementing the program, ethics committee approval was obtained from the İnönü University’s Social and Human Sciences Scientific Research and Publication Ethics Committee (10 May 2024, 10/15), and institutional permission was obtained from the Malatya Provincial Directorate of National Education (3 January 2025, MEB.TT.2024.013932). The research was conducted at a state nursery school affiliated with the Ministry of National Education (MoNE) in Turkey. The pre-school education environment in which the research was conducted was structured according to the MoNE Pre-school Education Program. The teachers in the classes where the study was conducted hold bachelor’s degrees in pre-school teaching and are actively engaged in pre-school teaching. The kindergarten where the research and implementation would be conducted was visited, and the school’s administrators and teachers were informed about the purpose and content of the study, the curriculum to be implemented, and the measurement tools to be used. Families were contacted, and information about the purpose and scope of the study, participation requirements, the time and duration of the measurements, the characteristics of the program to be implemented, the potential benefits and risks of participation, the principles of voluntariness, confidentiality, and the right to withdraw from the study at any time were provided through a written information form. Written informed consent was obtained from the parents after all questions were answered.

Before beginning the play-based mindfulness training program, pretest forms were administered to the experimental and control group students by their teachers. Twenty children in the experimental group participated in a play-based mindfulness training program comprising 20 sessions of approximately 40–45 min, twice a week for 10 weeks, while the 20 children in the control group attended only the standard preschool education program for the same duration. Sessions began with brief breathing and concentration exercises, followed by a play-based activity focused on the targeted skill and included brief reflection phases on the relationship between emotion, thought, and behavior. The measurement tools used in the study were administered to children in the experimental and control groups in two stages. Pre-test measurements were conducted in February 2025, and post-test measurements were conducted in May 2025, following the completion of the program implementation. All assessments were conducted by pre-school teachers who knew the children well; the measurements were applied in both groups at the same time intervals and under similar conditions. Due to the nature of the classroom-based intervention, teachers completing the outcome measures were not blinded to group allocation. After the training was completed, post-tests were administered to the experimental and control group students by their teachers.

### 2.4. Development and Implementation of the Play-Based Mindfulness Intervention Program

The Play-Based Mindfulness Training Program developed within the scope of this research was designed to support preschool children’s executive functions, emotion regulation skills, and resilience. The program is based on contemporary developmental theories and pedagogical approaches that center on the child’s multidimensional development. The program development process was conducted within the framework of the Taba-Tyler Program Development Model. The Taba–Tyler Program Development Model consists of complementary stages such as identifying needs, formulating objectives, content selection and organization, learning experiences, and evaluation [75].

*Identifying needs:* In the first step, a needs analysis was conducted with the teachers. For the needs analysis, the researcher prepared a semi-structured interview form to determine the teachers’ needs. The form was revised and finalized based on the opinions of two academics in child development and one academic in educational sciences. During the implementation process, the form was administered to 15 preschool teachers, and the obtained data were evaluated using content analysis. The analysis revealed that approximately 70% of the teachers expressed similar needs, and these findings were used as a basis for determining the program’s objectives and content according to the Taba–Tyler Model.

*Formulation of objectives:* In line with the identified needs, the general and specific objectives of the program were defined during the ‘formulation of objectives’ stage of the Taba–Tyler Program Development Model. The general objective of the program is to support preschool children’s emotional regulation, resilience, and executive function skills through play-based mindfulness activities. In line with this general objective, specific goals have been set to improve children’s social interaction, self-awareness, self-regulation, attention, and emotional awareness skills.

*Content selection and organization:* The program’s content is based on theoretical approaches grounded in the developmental characteristics of the preschool period. For example, Parten’s Social Play Theory emphasizes that children exhibit increased social interaction during play and that cooperative games are particularly important for social-emotional development. Within this theoretical framework, the program includes cooperative group games that support children’s interactive learning with their peers, as well as play activities designed to develop empathy, sharing, and turn-taking skills. This supports children in using their self-regulation and emotion regulation skills during social interaction. According to Fröbel, play is an activity in which children can express their inner world and themselves freely, and in which learning occurs naturally. The educational program developed based on Fröbel’s approach includes activities that support children’s creativity and symbolic play, which allows them to express their feelings and thoughts through play. These activities aim to help children make sense of their inner experiences and develop self-awareness [76]. Kabat-Zinn’s Mindfulness Approach is based on directing attention to the present moment in a non-judgmental and conscious manner [77]. In line with this, the educational program integrates body, breath awareness, and attention-focused activities with play-based activities, taking into account the developmental levels of children. These practices aim to support children’s ability to regulate their emotional responses, stay in the moment, and recognize their feelings.

In light of this institutional information and in line with the defined general and specific objectives, content selection and organization were carried out; a program draft was prepared by drawing on previously implemented awareness-based programs and the literature [64,78,79,80,81,82]. The prepared program draft was finalized based on the opinions of five experts (three academics in the field of child development, one academic in the field of special education, and one expert in the field of measurement and evaluation) to ensure the content’s suitability and consistency with the objectives. The program includes body, breath, movement, and sensory awareness (body awareness and perceiving the environment through the senses), emotional awareness and emotion management (recognizing and regulating basic emotions), regulating behavior and emotions (developing empathy, social adjustment, and positive social behavior), self-compassion (accepting oneself, asking for help, and developing compassion towards others), and attention and focus (selective attention, following instructions, problem-solving, and planning skills).

*Learning experiences:* In the ‘planning of learning experiences’ stage of the Taba–Tyler Program Development Model, the program was structured based on Vygotsky’s Sociocultural Theory. Group interaction-based games and emotional awareness games were included to enable children to learn through social interaction and develop their self-regulation skills [35]. Learning experiences were planned in line with the MEB (2023) Pre-school Education Program outcomes and indicators and adapted to the play-based learning approach. The program lasted for 10 weeks and was implemented in approximately 40 min sessions twice a week. Activity plans were prepared in line with the program objectives; materials were designed to be developmentally appropriate and safe; games and audio-visual tools were used in the activities, and large and small group discussions were included. Throughout the implementation, symbolic role-playing, breathing exercises, attention-focused games, and empathy activities were used to develop children’s ability to recognize their feelings, make non-judgmental observations, and stay in the moment. Children’s social-emotional skills, self-awareness, and self-regulation skills were supported through play-based learning with prompts such as ‘How is your breathing?’ and ‘How are you feeling right now?’

*Evaluation:* In the ‘evaluation’ phase of the Taba–Tyler Program Development Model, the program was evaluated in terms of its achievement of the defined objectives and its feasibility. To this end, prior to the program’s implementation, a pilot application was conducted in a preschool class not included in the study, accompanied by an expert observer. Necessary adjustments were made to the activity flow and program content based on the observations and feedback gathered during the pilot application. Measurements were taken before and after implementation to assess the program’s level of achievement of the targeted outcomes; changes in children’s resilience, executive functions, and emotion regulation skills were examined using valid and reliable measurement tools.

The program was specifically shaped based on Vygotsky’s Socio-Cultural Theory to help children learn about their emotions through social interactions and develop their self-regulation skills [35]. In this regard, emotional awareness games and group interactive games were included. In addition, games that develop empathy, patience, and cooperation in line with Parten’s Social Play Theory; free play and symbolic expressions within the framework of Froebel’s Play Theory were included in the program [76,83]. Kabat-Zinn’s Mindfulness Approach was utilized in the structure of the program, and children’s “present moment focus” and self-regulation skills were supported through breathing exercises, sensory awareness exercises, and body movements [77]. The program was structured by taking into account the MEB 2023 Pre-School Education Program achievement indicators and was aligned with the game-based learning approach.

### 2.5. Data Analyses

The SPSS 28 package program was used in data analysis. Before the analyses, the data were prepared, and it was determined that there were no missing or outlier values in the data, based on the criteria specified by Çokluk et al. (2021) [84]. Subsequently, the data were checked for normal distribution using skewness-kurtosis values, the Kolmogorov–Smirnov test, and histogram graphs. Accordingly, the skewness and kurtosis values of the scores falling between the +2 and −2 cut-off values and the appearance of the relevant histogram graphs indicate that the data were normally distributed in all tests for the groups (see Table 2). As a result of these analyses, it was decided to use parametric tests. Prior to the intervention, the pre-test scores of the experimental group and the control group were compared using an independent samples *t*-test. This analysis assessed pre-intervention group comparability by testing whether there were any statistically significant baseline differences in the study variables. Post-intervention pre-test and post-test scores for the study variables in the experimental and control groups were compared using a two-way analysis of variance (ANOVA) for mixed measures. Additionally, the Levene test was used to examine the homogeneity of variances, and significance scores were found to be greater than 0.05. Finally, eta-squared values (small = 0.01; medium = 0.06; large = 0.11) were calculated and interpreted for effect size estimates [85].

## 3. Results

### 3.1. Descriptive Statistics and Pre-Intervention Comparison Results

The analysis procedures were started by comparing the pre-test scores of the experimental and control groups (see Table 3). Accordingly, the CHEXI, DECA-P2, and ERS were administered to both groups before the intervention, and the resulting pre-test scores were analyzed using an independent samples *t*-test. The analyses revealed no statistically significant differences between the experimental and control groups in any of the subscales of the measurement tools (*p* > 0.05). These findings suggested that the experimental and control groups had similar characteristics in terms of executive functions, resilience, and emotion regulation skills before the intervention.

### 3.2. The Effect of Intervention on Resilience

The results of the two-way ANOVA for mixed measures to determine whether the changes observed in the DECA-P2’s subscales after the experiment were significantly different in the experimental and control groups are presented in Table 4. The results of the mixed effects model based on the groups and time interaction revealed that this interaction was significant in determining the effectiveness of the experimental intervention in the self-regulation subscale (Fgroups*time= 175.67; *p* = 0.00). The change in mean scores (Experimental post-test-pre-test = 7.85 > Control post-test-pre-test = 1.15) indicated that the increase occurred in the experimental group. Similarly, in the attachment subscale, the groups*time interaction showed that the experimental intervention was effective (Fgroups*time = 38.12; *p* = 0.00). The change in mean scores was larger in the experimental group (post-test–pre-test = 4.30) than in the control group (post-test–pre-test = 1.35), indicating that the experimental group showed greater improvement. Finally, the experimental intervention was also effective in increasing the initiative scores of the children in the experimental group (Fgroups*time = 27.54; *p* = 0.00). The change in mean scores was larger in the experimental group (post-test–pre-test = 2.90) than in the control group (post-test–pre-test = 0.15), indicating that the experimental group showed greater improvement. Estimated effect sizes were found to be large for the subscales of self-regulation (η^2^ = 0.82), attachment/relationship (η^2^ = 0.50), and initiative (η^2^ = 0.42).

### 3.3. The Effect of Intervention on Emotion Regulation Skills

The results of the two-way ANOVA for mixed measures to determine whether the changes observed in the experimental and control groups after the experiment in the ERS’s subscales are significant are presented in Table 5. The mixed effects model results show that the groups*time interaction was significant in the negativity subscale (Fgroups*time = 35.13; *p* = 0.00). The reduction in mean scores was greater in the experimental group (pre-test–post-test = 6.6) than in the control group (pre-test–post-test = 2.5), indicating a larger improvement in the experimental group. Similarly, the group*time interaction was significant in the emotion regulation subscale (Fgroups*time = 20.78; *p* = 0.00). The increase in mean scores was larger in the experimental group (post-test–pre-test = 3.75) than in the control group (post-test–pre-test = 1.45), indicating that the experimental group showed greater improvement. Estimated effect sizes were found to be large for the negativity (η^2^ = 0.48) and emotion regulation (η^2^ = 0.35) subscales.

### 3.4. The Effect of Intervention on Executive Functions

A two-way ANOVA for mixed measures was used to compare the changes observed after the experiment in the CHEXI’s subscales of the children in the experimental and control groups to determine whether there were significant differences. The results are presented in Table 6. Accordingly, repeated measurements (pre-test and post-test) performed in the experimental and control groups showed a statistically significant effect on the children’s working memory levels. The results of the mixed effects model based on the groups and time interaction revealed that this interaction was significant in determining the effectiveness of the experimental intervention (Fgroups*time = 101.1; *p* = 0.00). The change in the mean scores in the working memory subscale (Experimental post-test-pretest = 13.95 > Control post-test-pretest = 1.55) indicated that the increase occurred in the experimental group. Similarly, the groups*time interaction in the inhibition subscale presented that the experimental intervention was effective (Fgroups*time = 101.47; *p* = 0.00). The change in mean scores on the inhibition subscale (Experimental post-test-pre-test = 9.85 > Control post-test-pre-test = 1.70) indicated an increase in the experimental group. Estimated effect sizes were large for working memory (η^2^ = 0.72) and inhibition (η^2^ = 0.72).

## 4. Discussion

The primary aim of this study was to examine the effects of a play-based mindfulness training program on the resilience, emotion regulation and executive functions of preschool children aged 5–6 years. The findings revealed that children in the experimental group showed significant improvements in three areas compared to the control group. In particular, the intervention was associated with an increase in protective factors related to resilience, improvements in working memory and inhibition skills, a decrease in negativity, and better emotion regulation strategies.

This study is not limited to reporting child-level outcomes; it also draws attention to the determining role of program design in the effectiveness of mindfulness-based interventions during early childhood. The intervention program was developed on the basis of the Taba–Tyler curriculum development model and was structured in line with needs analyses obtained from teachers and feedback from experts, thereby achieving a high degree of congruence with the developmental characteristics of preschool children. Whereas many studies in the literature employ simplified adaptations of mindfulness protocols originally designed for adults [12,23], the program developed in the present study was designed within a pedagogically coherent framework aligned with the preschool curriculum and was needs-based. These features are regarded as key factors that support both the sustainability and the effectiveness of the intervention.

### 4.1. Resilience

In the current study, it was determined that changes in self-regulation, attachment/relationship, and initiative were more favorable in the experimental group than in the control group. This pattern reflects stronger resilience-related functioning in the experimental group in terms of protective characteristics. Luthar, Cicchetti, and Becker (2000) stated that programs structured with social support systems play a critical role in developing children’s resilience [40]. In this context, the program implemented in the current study aimed to develop resilience by emphasizing games that increase social interaction. Bazzano et al. (2023), in a mindfulness- and yoga-based program implemented with children aged 3–5 years, reported significant improvements in children’s protective factors associated with psychological resilience and in their social–emotional competencies [43]. Similarly, the mindfulness- and yoga-based social–emotional learning program developed by Courbet et al. (2022) was characterized as an intervention model that supports psychological well-being in preschool children [86]. Collectively, these studies suggest that mindfulness-based approaches in the preschool period may strengthen developmental domains related to psychological resilience. In these programs [43,86], yoga and mindfulness practices were predominantly delivered through activities emphasizing bodily awareness and individual self-regulation. By contrast, in the present study, mindfulness content was developed based on play types derived from Parten’s play theory (e.g., parallel play and cooperative play), thereby integrating play processes that are developmentally attuned to children’s levels of social interaction into the program. This approach enabled children not only to cultivate individual mindfulness-related awareness skills but also, through peer interaction, to experience and further develop emotional and social protective factors associated with psychological resilience.

Fyffe and Lewis (2024) demonstrated that, during the pandemic, play-based learning in preschool children facilitated coping with everyday challenges and supported processes of emotional adjustment [87]. This finding parallels the resilience gains observed in the present study. In another study, Treves et al. (2023) reported that naturally developing tendencies toward dispositional mindfulness over time enhanced children’s emotional resilience in the face of stressful life events [88]. Likewise, Laundy et al. (2021) found that a mindfulness-based intervention implemented with primary and middle school students had a positive long-term effect on children’s resilience [89]. Taken together, these findings are consistent with the present study’s results, indicating that the program delivered to children strengthened their resilience-related skills.

### 4.2. Emotion Regulation Skills

When examining the changes in the students’ negativity and emotion regulation subscale scores, it was determined that the increase in the mean scores of the experimental group was more significant than that of the control group. This pattern indicates that children in the experimental group showed more favorable change in noticing, recognizing, expressing, and regulating emotional reactions, including coping with emotions such as anger, sadness, and frustration. These findings are consistent with studies in the literature. For example, Zelazo and Lyons (2012) emphasized the important role that mindfulness training plays in developing self-regulation skills in early childhood [8]. A study by Dreimane and Vītola (2024) indicated that mindfulness practices in early childhood education may help children develop more balanced responses to emotional intensities [90]. A systematic review by Rowland, Hindman, and Hassmén (2023) evaluated 15 different mindfulness-based group interventions and concluded that they generally had positive effects on emotion regulation, attention, and body awareness [91]. However, attention was drawn to the scale distribution, and it was stated that the results should be interpreted with caution [91]. This makes the current study stand out in terms of the validity, reliability, and age-appropriateness of the measurement tool used. Hanceroglu (2017) reported that younger children in the mindfulness group showed significant improvements in working memory and cognitive flexibility skills, while older children experienced a significant decrease in rumination [92]. This age-related variation suggests that mindfulness-related outcomes may present differently across developmental periods, and evidence from mixed-age samples should be interpreted carefully when applied to preschool children. Although systematic reviews and meta-analyses covering broad age ranges (e.g., Dunning et al., 2019; Rowland et al., 2023; Laundy et al., 2021) provide robust evidence for the effectiveness of mindfulness practices, it should be noted that the samples in a substantial proportion of these studies predominantly comprise adolescents [11,89,91]. Therefore, the direct generalization of findings derived from mindfulness interventions designed for older age groups to the preschool period is limited, and such results should be interpreted with caution. Whereas mindfulness in adolescents is often operationalized through processes such as introspective observation, cognitive restructuring, and abstract thinking, these capacities are still in the process of developmental maturation in preschool children aged 5–6 years. Accordingly, in preschool-focused interventions, concrete and play-based tools are employed rather than extended periods of silent meditation typically used with adolescents. For instance, program activities incorporated tangible materials such as plush toys placed on the abdomen to support breathing awareness and emotion cards used to visualize feelings. This implementation strategy transformed mindfulness training from a static practice into an interactive experience in which children received feedback through concrete objects and engaged actively. When interpreting the preschool findings by extrapolating from adult and adolescent research, it is essential to recognize that these developmental differences directly shape both the form of the intervention (i.e., its play-based nature) and the outcomes that can reasonably be expected. The positive findings obtained in the present study indicate that the overall efficacy of mindfulness reported in the literature may also be realized in younger age groups, such as preschoolers, when supported by developmentally appropriate pedagogical methods and gamified intervention components.

Furthermore, Yıldırım (2022) reported positive improvements in children’s empathy, recognition of emotions, and appropriate emotional responses through social interactions in a mindfulness-based peer relationship program he developed for preschool children [81]. This research supports the contribution of the play-based mindfulness activities used in the current study to social and emotional functioning through the natural learning process created through play. As a result of the implementation of the game-based programme developed by Öztekin (2024) for primary school students, children experienced an increase in empathy, prosocial behaviors and emotional awareness, thus indirectly supporting emotion regulation skills [93]. Similarly, Khazaeili et al. (2023) reported that a family-centered mindfulness-based play program increased emotion regulation skills by reducing negative emotional reactions in children [94]. Taken together, these findings support the interpretation that mindfulness-oriented content may be particularly compatible with early childhood learning when delivered in a play- and interaction-based format.

In conclusion, the play-based mindfulness program implemented in this study appears to have improved children’s emotion regulation skills, with similar results to the current study. Throughout the program, activities focused on helping children identify, name, express, and appropriately manage their emotions. These activities encouraged children to accept their negative emotions without suppressing them, thereby supporting their ability to cope with them in healthy ways. In this respect, the current study aligns with existing literature, which emphasizes that mindfulness-based approaches to helping children regulate their emotions are more effective when presented through play, and aligns with the theoretical foundations in the field.

### 4.3. Executive Functions

The experimental group showed greater improvement in working memory and inhibition than the control group. Executive functions encompass higher-level cognitive processes that develop rapidly in early childhood, such as cognitive flexibility, attentional control, inhibition, problem solving, and planning [18]. In particular, supporting skills such as controlling impulsive reactions, increasing attentional focus, monitoring thoughts, and responding flexibly to situations play a critical role in the development of executive functions in early childhood. Diamond and Lee (2011) highlighted the supportive effect of structured play implemented in the preschool period on executive functions, stating that structured, rule-based, and instruction-based games, in particular, improve children’s inhibition and attention control, and contribute to creative problem-solving and cognitive flexibility skills [95]. In this context, the attention-focusing games, instruction-following activities, patient waiting, and turn-taking-based activities included in the training program developed in our study effectively supported children’s cognitive processes. Similarly, Newton (2022) stated that structured play-based learning environments naturally support executive functions; Gibb et al. (2021) stated that play-based programs improve preschool children’s self-regulation skills and cognitive flexibility [96,97]. A study by Yaffe et al. (2025) among primary school students found that even short-term game-based interactions led to significant improvements in executive function skills [98]. Together, these studies support the view that combining mindfulness-oriented content with structured play may be developmentally aligned with executive-function gains in preschool children.

In Aydın’s (2022) study, it was reported that a mindfulness-based education program led to significant improvements in children’s executive functions, particularly in the areas of sustained attention, impulse control, and planning [64]. These findings are consistent with the results of the present study. However, unlike the study by Aydın (2022), the targeted learning outcomes in our study are more explicitly integrated with Vygotsky’s Sociocultural Theory and Fröbel’s play pedagogy. Accordingly, this study addressed mindfulness-related skills not as independent cognitive tasks to be applied in isolation, but by embedding them within play, which is the child’s natural form of learning and communication. This facilitated the internalization of mindfulness through developmentally appropriate experiences. In this respect, the present study differs from the existing literature by positioning mindfulness within a play-based pedagogical framework rather than applying it primarily as a technically focused educational format.

In addition, Atalay (2018) stated that mindfulness practices directly affect attention and self-regulation processes and improve an individual’s ability to stay mentally present “here and now” [99]. This skill is important for children to direct their attention to a task, recognize and recover distractions, and demonstrate patience in problem-solving processes. Activities involving strategies such as the “mindful listening game” and “stop yourself-think-apply” within the program are thought to directly target subcomponents of executive functions (especially inhibition and cognitive flexibility). Shlomov et al. (2023) confirmed at the neural level that a curriculum based on mindfulness and kindness had a significant effect on executive function components, particularly inhibition, in preschool children [10]. Furthermore, Xie et al. (2024) demonstrated that a mindfulness-based intervention produced functional changes in some brain regions associated with executive functions in children, and that these changes increased the efficiency of cognitive processes [100]. The improvements observed in the present study are consistent with this body of work linking mindfulness-based approaches with executive-function-related outcomes across behavioral and neural indicators.

The results demonstrated that mindfulness practices implemented through individual or group-based play enhance the development of executive function skills and allow children to experience cognitive skills in natural learning environments. The use of play in activities designed to develop executive function components, such as turn-taking, attention focus, problem solving, following instructions, and alternative thinking, facilitated the internalization of cognitive processes and contributed to increased attention spans, inhibition of inappropriate responses, and improved task retention skills. In these respects, the executive function skills fostered by the program align with the literature demonstrating that play-based mindfulness practices in early childhood contribute positively to executive functions.

## 5. Limitations

The findings of this study indicate that the play-based mindfulness intervention program had positive effects on resilience, emotion regulation, and executive function skills among preschool children. Nevertheless, these results should be interpreted in light of several limitations of the study. In this study, the game-based mindfulness program delivered to the experimental group provided children with structured and planned learning experiences. Accordingly, when interpreting the findings, it should be considered that the observed improvements may reflect not only the program’s content but also the effects of children’s participation in such regular, salient/engaging activities. Another limitation of the study concerns the assessment of resilience. In the preschool and early childhood period, resilience is typically evaluated through indicators related to social–emotional development. However, it should be acknowledged that such indicators may not fully capture all dimensions of the construct. Therefore, future research is needed to address resilience in a more comprehensive and clearly delineated manner by incorporating different measurement tools.

In this study, resilience, emotion regulation, and executive functions were examined concurrently; however, the relationships among these skills were not statistically tested. Although this is consistent with the primary aims of the research, it highlights an important direction for future studies. Another limitation is that the participants were limited to children aged 5–6 years, which may constrain the generalizability of the findings to other age groups. In addition, the control group continued with standard preschool education without receiving any intervention. While this approach is consistent with comparable experimental studies in the field, future research could benefit from incorporating alternative comparison groups, which may allow the program’s effects to be delineated in greater detail and yield more comprehensive conclusions. Finally, as the results were assessed using teacher-rated questionnaires and teachers were not blinded to group allocation, the findings may be subject to expectancy bias. Future studies could strengthen internal validity by using blinded independent assessors and incorporating objective measures alongside teacher reports.

## 6. Conclusions and Recommendations

This study was conducted to examine the effects of a play-based mindfulness training program on preschool children’s resilience, emotion regulation, and executive functioning skills. The findings indicated that the play-based mindfulness training program significantly improved the children in the experimental group’s resilience (improvements in self-regulation, attachment/relationship, and initiative), emotion regulation (emotion regulation and negativity), and executive function skills (working memory and inhibition). At the end of the program, the children in the experimental group demonstrated higher levels of emotional awareness compared to the control group, were able to cope more effectively with challenging emotions such as anger and stress, and were more successful in skills such as self-expression, asking for help, and maintaining social relationships. In the area of executive functions, cognitive skills such as attention control, sequencing, following instructions, and problem-solving were improved, and play-based practices were understood to help structure children’s mental processes. In the area of emotion regulation, children were observed to make progress in recognizing, naming, and appropriately expressing their emotions, and to internalize emotion regulation strategies. When looked at from the perspective of resilience, it was concluded that children can cope more calmly and positively when faced with stressful situations, develop self-awareness, and maintain their social relationships more robustly.

These results demonstrated that play-based mindfulness programs can be a powerful tool for the multifaceted development of preschool children. In early childhood, considered the most critical period of life, providing structured mindfulness programs based on play may both make learning enjoyable and enhance cognitive and emotional flexibility.

Research findings indicate the applicability and potential contributions of game-based mindfulness programs and approaches structured on this basis in pre-school educational settings. In this context, it is recommended that such mindfulness programs, like the one developed in this study, be implemented by integrating them with play, because play is the child’s most natural learning tool in the preschool period, enabling the concretization of abstract concepts, ensuring active participation, and facilitating the internalization of learned skills. Moreover, it is recommended that families also be involved in the process to ensure the sustainability of program gains. Parent seminars, home implementation forms, and shared activity guides can be developed to ensure permanence in children’s behavior. Because the study only covered the 5–6 age group, versions of the program adapted for different age levels and children with developmental differences can be developed. Additionally, future research could incorporate qualitative data collection tools (teacher journals, children’s drawings, etc.) to provide a more in-depth understanding of the program’s effectiveness and experiences.

## Figures and Tables

**Table 1:** Frequency and percentage distributions of demographic characteristics of participants in the experimental and control groups.

Characteristics		Groups					
		Control		Experimental			
		n	%	n	%	*χ^2^*	*p*
Gender	Female	10	50.0	9	45.0	0.100	0.752
	Male	10	50.0	11	55.0		
Age	Five	9	45.0	13	65.0	1.616	0.204
	Six	11	55.0	7	35.0		
Number of children	1	5	25.0	6	30.0	2.424	0.489
	2	10	50.0	11	55.0		
	3	5	25.0	2	10.0		
	4 or more	0	0.0	1	5.0		
Whether the mother is living	Yes	20	100	20	100	-	-
	No	0	0.0	0	0.0		
Whether the father is living	Yes	20	100	20	100	-	-
	No	0	0.0	0	0.0		
Whether the parents are living together	Yes	20	100	20	100	-	-
	No	0	0.0	0	0.0		
Educational level of the mother	High school	1	5.0	1	5.0	0.543	0.762
	University	13	65.0	15	75.0		
	Postgraduate	6	30.0	4	20.0		
Educational level of the father	High school	0	0.0	2	10.0	2.286	0.319
	University	12	60.0	12	60.0		
	Postgraduate	8	40.0	6	30.0		
Occupation of the mother	Housewife	1	5.0	3	15.0	3.000	0.392
	Civil servant	15	75.0	10	50.0		
	Worker	1	5.0	1	5.0		
	Other	3	15.0	6	30.0		
Occupation of the father	Civil servant	14	70.0	10	50.0	3.333	0.189
	Worker	1	5.0	0	0.0		
	Other	5	25.0	10	50.0		

**Table 2:** Results of the reliability and normality analyses of the scales.

Scales	Groups	Tests	Cronbach’s Alpha (α)	Skewness	Kurtosis
Working Memory	Experimental	Pre-test	0.842	0.605	0.530
		Post-test	0.864	0.672	−0.496
		Continuance	0.869	0.658	−0.362
	Control	Pre-test	0.805	0.371	0.077
		Post-test	0.867	0.445	0.448
Inhibition	Experimental	Pre-test	0.846	0.783	−0.039
		Post-test	0.821	0.277	−0.443
		Continuance	0.885	0.440	−0.857
	Control	Pre-test	0.857	0.430	−0.685
		Post-test	0.848	0.506	−0.643
Self-regulation	Experimental	Pre-test	0.788	−0.978	0.165
		Post-test	0.769	−0.417	−0.570
		Continuance	0.751	−0.784	0.155
	Control	Pre-test	0.742	0.273	−0.828
		Post-test	0.738	0.082	−0.708
Attachment/Relationship	Experimental	Pre-test	0.801	0.712	−0.200
		Post-test	0.798	0.562	0.294
		Continuance	0.776	1.247	1.067
	Control	Pre-test	0.768	0.413	0.034
		Post-test	0.787	0.384	−0.545
Initiative	Experimental	Pre-test	0.764	−1.194	0.680
		Post-test	0.791	−1.252	0.849
		Continuance	0.803	−0.559	1.632
	Control	Pre-test	0.754	−1.247	1.429
		Post-test	0.784	−1.483	1.617
Negativity	Experimental	Pre-test	0.807	1.760	1.134
		Post-test	0.788	1.374	0.975
		Continuance	0.704	1.012	1.878
	Control	Pre-test	0.708	0.353	−0.673
		Post-test	0.718	0.226	−0.940
Emotion Regulation	Experimental	Pre-test	0.710	−0.101	−0.758
		Post-test	0.724	−0.096	−0.716
		Continuance	0.798	−0.160	−0.518
	Control	Pre-test	0.762	−0.219	0.104
		Post-test	0.732	−0.680	0.776

**Table 3:** Comparison of the pre-test scores of the experimental and control groups.

Scales	Subscales	Groups	n	X̄	SE	sd	*t*	*p*
CHEXI	Working Memory	Experimental	20	27.90	8.819	38	0.394	0.696
		Control	20	26.80	8.859			
	Inhibition	Experimental	20	32.40	6.557	38	0.585	0.562
		Control	20	31.05	7.970			
		Control	20	30.55	4.298			
DECA-P2	Self-regulation	Experimental	20	23.95	4.136	38	0.664	0.511
		Control	20	24.80	3.955			
	Attachment/Relationship	Experimental	20	26.30	1.689	38	1.259	0.216
		Control	20	25.45	2.502			
	Initiative	Experimental	20	26.30	2.494	38	0.651	0.519
		Control	20	25.65	3.703			
ERS	Negativity	Experimental	20	27.55	5.987	38	0.816	0.420
		Control	20	26.20	4.348			
	Emotion Regulation	Experimental	20	20.60	2.836	38	0.835	0.409
		Control	20	21.20	1.508			

**Table 4:** Two-Factor ANOVA (Mixed Measures) results of the DECA-P2’s subscales.

	Experimental Group		Control Group					
	Pre-Test Mean ± SD	Post-Test Mean ± SD	Pre-Test Mean ± SD	Post-Test Mean ± SD		Groups	Time	Groupsxtime
Self-regulation	23.95 ± 4.13	31.80 ± 3.59	24.80 ± 3.95	25.95 ± 3.92	F-value	4.27	316.99	175.67
					*p*	0.04	0.00	0.00
					η^2^	0.10	0.89	0.82
Attachment/Relationship	26.3 ± 1.68	30.60 ± 1.46	25.45 ± 2.50	26.80 ± 2.41	F-value	14.58	139.83	38.12
					*p*	0.00	0.00	0.00
					η^2^	0.27	0.78	0.50
Initiative	26.30 ± 2.49	29.20 ± 2.14	25.65 ± 3.70	25.80 ± 4.00	F-value	4.33	33.87	27.54
					*p*	0.04	0.00	0.00
					η^2^	0.10	0.47	0.42

**Table 5:** Two-Factor ANOVA (mixed measures) results of the ERS subscales.

	Experimental Group		Control Group					
	Pre-Test Mean ± SD	Post-Test Mean ± SD	Pre-Test Mean ± SD	Post-Test Mean ± SD		Group	Time	Groupxtime
Subscales of the ERS								
Negativity	27.55 ± 5.98	20.95 ± 4.60	26.20 ± 4.34	23.70 ± 3.58	F-value	0.23	173.09	35.13
					*p*	0.63	0.00	0.00
					η2	0.00	0.82	0.48
Emotion Regulation	20.60 ± 2.83	24.35 ± 2.27	21.20 ± 1.50	22.65 ± 2.18	F-value	0.68	106.25	20.78
					*p*	0.41	0.00	0.00
					η2	0.01	0.73	0.35

**Table 6:** Two-Factor ANOVA (mixed measures) results of CHEXI’s subscales.

	Experimental Group		Control Group					
	Pre-Test Mean ± SD	Post-Test Mean ± SD	Pre-Test Mean ± SD	Post-Test Mean ± SD		Groups	Time	Groupsxtime
Subscales of the CHEXI								
Working Memory	27.90 ± 8.81	41.85 ± 9.88	26.80 ± 8.85	28.35 ± 8.79	F-value	6.74	157.97	101.1
					*p*	0.01	0.00	0.00
					η^2^	0.15	0.80	0.72
Inhibition	32.40 ± 6.55	42.25 ± 7.34	31.05 ± 7.97	32.75 ± 7.64	F-value	5.54	203.79	101.47
					*p*	0.02	0.00	0.00
					η^2^	0.12	0.84	0.72

## Data Availability

The Dataset is available on request from the authors. The data are not publicly available confidentiality considerations.
